# Evaluation of Alpha-1 Antitrypsin Levels and *SERPINA1* Gene Polymorphisms in Sickle Cell Disease

**DOI:** 10.3389/fimmu.2017.01491

**Published:** 2017-11-06

**Authors:** Magda Oliveira Seixas Carvalho, André Luís Carvalho Santos Souza, Mauricio Batista Carvalho, Ana Paula Almeida Souza Pacheco, Larissa Carneiro Rocha, Valma Maria Lopes do Nascimento, Camylla Vilas Boas Figueiredo, Caroline Conceição Guarda, Rayra Pereira Santiago, Adekunle Adekile, Marilda de Souza Goncalves

**Affiliations:** ^1^Instituto Gonçalo Moniz-Fiocruz-Bahia (IGM-FIOCRUZ-Ba), Salvador, Brazil; ^2^Complexo Hospitalar Universitário Professor Edgard Santos, Salvador, Brazil; ^3^Universidade Federal da Bahia (UFBA), Salvador, Brazil; ^4^Fundação de Hematologia e Hemoterapia da Bahia, Salvador, Brazil; ^5^Department of Pediatrics, Kuwait University, Kuwait City, Kuwait

**Keywords:** sickle cell disease, alpha-1 antitrypsin, *SERPINA1*, biomarkers, inflammation

## Abstract

Alpha-1 antitrypsin (AAT) is an inhibitor of neutrophil elastase and a member of the serine proteinase inhibitor (serpin) superfamily, and little is known about its activity in sickle cell disease (SCD). We hypothesize that AAT may undergo changes in SCD because of the high oxidative stress and inflammation associated with the disease. We have found high AAT levels in SCD patients compared to controls, while mutant genotypes of *SERPINA1* gene had decreased AAT levels, in both groups. AAT showed negative correlation with red blood cells, hemoglobin (Hb), hematocrit, high-density lipoprotein cholesterol, urea, creatinine, and albumin and was positively correlated with mean corpuscular Hb concentration, white blood cells, neutrophils, Hb S, bilirubin, lactate dehydrogenase, ferritin, and C-reactive protein. Patients with higher levels of AAT had more infection episodes (OR = 1.71, CI: 1.05–2.65, *p* = 0.02), gallstones (OR = 1.75, CI: 1.03–2.97, *p* = 0.02), and had more blood transfusions (OR = 2.35, CI: 1.51–3.65, *p* = 0.0001). Our data on AAT association with laboratory indices of hemolysis and inflammation suggest that it may be positively associated with SCD severity; the negative correlations with renal parameters suggest a cytoprotective mechanism in SCD patients. In summary, AAT may need to be included in studies related to SCD and in the discussion of further therapeutic strategies.

## Introduction

Clinical symptoms associated with sickle cell disease (SCD) are heterogeneous, with the presence of hemolytic anemia, vaso-occlusive events, infections, acute chest syndrome (ACS), pulmonary hypertension, stroke, and glomerulopathy, among others (1–5). SCD has several sub-phenotypes and the search for biomarkers related to the disease severity is very useful to patients’ follow-up (6, 7).

Alpha-1 antitrypsin (AAT) is a glycoprotein of 52 kDa with 394 amino acids that is secreted and synthesized primarily not only in hepatocytes, but also on phagocytic cells, such as neutrophils, monocytes, and macrophages; lung epithelial cells; and intestinal cells. It is considered an acute phase protein, but it is also known as a hepatic stress protein, since its plasma levels increase during inflammation or tissue injury, and are related to inhibition of proteases that trigger inflammatory reactions (8). AAT controls the tissue degradation promoted by proteases, especially elastase, since it inhibits the pro-inflammatory action of these enzymes on specific tissues, such as lung as well as in neutrophils (9–11).

Alpha-1 antitrypsin is encoded by the *SERPINA1* gene that is located in the protease inhibitor locus, and there are more than 500 single-nucleotide polymorphisms described on this gene. Some of them are related to AAT expression changes and also with hepatic damage due to the retention of protein in hepatocytes, and occurrence of thrombosis, liver disease, pulmonary edema, emphysema, and chronic obstructive pulmonary disease (COPD) (11–17).

The *SERPINA1* gene is highly polymorphic (10, 18, 19). The wild-type allele is designated proteinase inhibitor (PI)*M, and the PI*S and PI*Z alleles are associated with AAT deficiency (16). Homozygotes for the PI*Z allele have about 15% of normal levels of AAT and have an increased risk for developing emphysema and to a lesser extent, liver disease in neonates. Heterozygotes for PI*MZ allele express about 60% of normal levels of AAT of MM homozygotes, whereas PI*MS heterozygotes express about 80% of normal levels of AAT (10, 16, 20).

Although AAT levels have been sporadically investigated in some reports on SCD, this protein was not considered to be a promising biomarker and was found to be only correlated with disease severity (21–24). In addition, another study investigated associations between biochemical genotypes and the clinical course of SCD, but did not attempt to establish correlations with specific genetic genotypes (25).

The present study tests the hypothesis that AAT may have changed its function in SCD patients, since they are subjected to prolonged oxidative stress and inflammatory conditions.

## Materials and Methods

### Casuistic

A total of 356 steady-state, unrelated SCD patients (235 HbSS, 115 HbSC, and 5 HbSβ^+^) were included in the present study. The patients’ mean (±SD) age was 13.96 ± 9.91 years, with a median of 12.00, 25th percentile of 8.00, and 75th percentile of 16.00 years, and 46% (165/356) were females. All patients were followed (2010–2014) at the outpatient pediatric hematology unit of the Bahia Hematology and Hemotherapy Foundation (HEMOBA). All patients were in steady state, i.e., none had received a blood transfusion 4 months prior to inclusion and no acute events, hospitalization, or infections were reported 3 months prior to blood sampling. No patients had taken antibiotics, hydroxyurea, or corticosteroids 10 days prior to blood sampling, but some were receiving folic acid therapy. Blood samples were taken during a regular clinical visit, and each patient’s medical history was obtained from patient records.

The control group consisted of 132 unrelated healthy individuals without any clinical or biochemical evidence of SCD; their mean age was 9.96 ± 3.17 years, and 48.5% (64/132) were female. These individuals were matched for sex and age with the SCD patients and were recruited from the geographic region.

The present study received approval from the Institutional Review Board of the Gonçalo Moniz Institute of the Oswaldo Cruz Foundation (IGM-FIOCRUZ), and each included study subject’s legal guardian agreed to participation and biological sample collection. This study followed the ethical guidelines established by the Declaration of Helsinki, as well as its subsequent revisions, and informed written consent was obtained from each control subject and SCD patient’s guardian. When applicable, the children’s acceptance was registered.

### Laboratory Characterization

Biological sample analysis was performed at the Laboratory of Hematology, Genetic and Computational Biology (LHGB) at IGM-FIOCRUZ and at the Clinical Analysis Laboratory of the School of Pharmacy (LACTFAR) at the Federal University of Bahia (UFBA).

Hematological analyses were performed by automated ABX Pentra 80 hematology analyzer (HORIBA DIAGNOSTICS, Montpellier, France) and blood smears were stained with Wright’s stain and examined by light optical microscopy. Reticulocytes were counted after staining with brilliant cresyl blue supravital dye (26). Hemoglobin (Hb) profiles were confirmed by high-performance liquid chromatography (Bio-Rad Variant-I; Bio-Rad, Hercules, CA, USA).

Liver, renal, lipid, inflammation, and hemolysis profiles, including AAT and ferritin serum concentration, were analyzed using an automated A25 Random Access Analyzer (Biosystems S.A, Costa Brava, Barcelona) and an Access 2 Immunoassay System with an IMMAGE Immunochemistry System (Beckman Coulter, Inc., Fullerton, CA, USA).

### Molecular Investigation

Genomic DNA was extracted from peripheral leukocytes using a Flexigene DNA Kit (QIAGEN Inc., Valencia, CA, USA) and quantified by spectrophotometry (Nanodrop^®^ ND-1000, NanoDrop Technologies, Inc., Wilmington, NC, USA). *SERPINA1* gene variants were investigated in randomly selected patients and controls by duplex polymerase chain reaction (PCR) using a combination of specific primers for the detection of allele variants PI*M, PI*S, and PI*Z in a single reaction, followed by the digestion of PCR products with *TaqI* restriction enzyme (27).

### Statistical Analysis

Statistical analyses were performed using EPI INFO software version 6.04, SPSS version 18.0, and GraphPad version 5.0. *p* Values <0.05 were considered significant. Quantitative variables with normal distribution were analyzed using the Kolmogorov–Smirnov test. Parametric ANOVA was used to analyze the means of quantitative or numerical variables with normal distributions, while the nonparametric Kruskal–Wallis test was used for data with non-normal distribution. When multiple comparisons were made, mean values and averages with significant differences were verified by Bonferroni correction.

The analysis of qualitative or categorical variables with two or more categories was performed by non-parametric chi-square (χ^2^) test, corrected by Mantel–Haenszel and Yates tests. Fisher’s exact test was used to perform comparisons among fewer than four categorical variables. A confidence interval of 95% was assumed, and odds ratios were calculated.

The independent *T*-test and Mann–Whitney test were used to assess differences in means between two unrelated groups within a variable, taking into account the distribution of each variable.

## Results

### Patient and Control Group Characteristics

Table 1 shows the hematological and biochemical laboratory parameters of SCD patients, with results presented as mean ± SD.

**Table 1:** Hematological and biochemical laboratory parameters of patients with sickle cell disease.

Laboratory values	*N*	Mean	SD	Percentile values		
				25th	50th	75th
RBC, ×10^12^/mL	355	3.25	0.93	2.53	3.00	4.00
Hemoglobin, g/dL	356	9.35	1.95	8.00	9.00	11.00
Hematocrit, %	356	27.18	6.00	22.13	26.00	32.00
MCV, fL	356	85.62	9.21	79.15	85.60	92.00
MCH, ρg	356	29.62	3.78	27.00	29.65	32.00
Reticulocyte count, %	352	6.07	2.58	4.00	6.00	7.88
Fetal hemoglobin (Hb), %	356	7.39	6.57	2.00	5.35	11.00
S Hb, %	356	72.63	17.95	51.32	81.00	88.35
Leukocyte count, ×10^9^/mL	356	11,955.31	4,180.38	8,800.00	11,450.00	14,600.00
Neutrophil count, ×10^9^/mL	356	5,891.08	2,789.25	3,870.00	5,357.00	7,391.00
Eosinophil count, ×10^9^/mL	356	768.17	712.07	274.00	521.50	1,109.75
Lymphocyte count, ×10^9^/mL	356	4,327.80	2,027.55	2,924.50	3,922.00	5,229.25
Monocyte count, ×10^9^/mL	356	826.21	402.89	537.00	741.00	1,070.75
Platelet count, ×10^3^/μL	356	407.21	158.74	288.50	392.00	507.50
Glucose, mg/dL	356	75.14	19.72	68.00	74.00	79.00
Total cholesterol, mg/dL	356	130.25	28.39	110.00	126.50	147.00
HDL-C, mg/dL	356	33.21	8.73	27.00	32.00	38.00
LDL-C, mg/dL	356	77.88	24.35	62.00	76.00	92.00
VLDL-C, mg/dL	356	19.23	9.73	13.00	17.00	23.00
Triglycerides, mg/dL	356	96.18	48.47	64.00	86.50	116.00
ALT, U/L	356	22.99	14.72	14.00	20.00	27.00
AST, U/L	356	47.68	22.13	32.00	43.00	60.00
Iron serum, mcg/dL	356	91.67	51.02	60.00	82.00	105.75
Ferritin, ηg/mL	351	305.68	458.89	81.00	167.20	320.60
Total bilirubin, mg/dL	356	1.99	1.35	1.00	1.90	2.50
Direct bilirubin, mg/dL	356	0.51	0.47	0.00	0.40	1.00
Indirect bilirubin, mg/dL	356	1.49	1.23	0.90	1.00	2.00
Total protein, g/dL	356	7.96	0.80	7.50	8.00	8.30
Albumin, g/dL	356	4.45	0.54	4.00	4.40	5.00
Globulin, g/dL	356	3.54	0.82	3.00	3.55	4.00
Uric acid, mg/dL	356	4.12	1.27	3.00	4.00	5.00
Urea nitrogen, mg/dL	356	18.17	8.07	13.00	17.00	21.00
Creatinine, mg/dL	354	0.54	0.51	0.00	1.00	1.00
C-reactive protein, mg/L	353	6.74	9.57	2.47	4.00	7.00
Antistreptolysin-O, UI/mL	353	177.68	339.38	28.50	80.00	165.00
Haptoglobin, mg/dL	353	8.82	15.75	5.83	6.00	6.00
LDH, U/L	356	964.05	531.42	597.25	855.00	1199.75

*RBC, red blood cell; MCV, mean cell volume; MCH, mean cell hemoglobin; HDL-C, high-density lipoprotein cholesterol; LDL-C, low-density lipoprotein cholesterol; VLDL-C, very low-density lipoprotein cholesterol; AST, aspartate aminotransferase; ALT, alanine aminotransferase; LDH, lactate dehydrogenase*.

### Differential Levels of AAT Are Associated with Different Genotypes of *SERPINA1*

**Figure 1:**
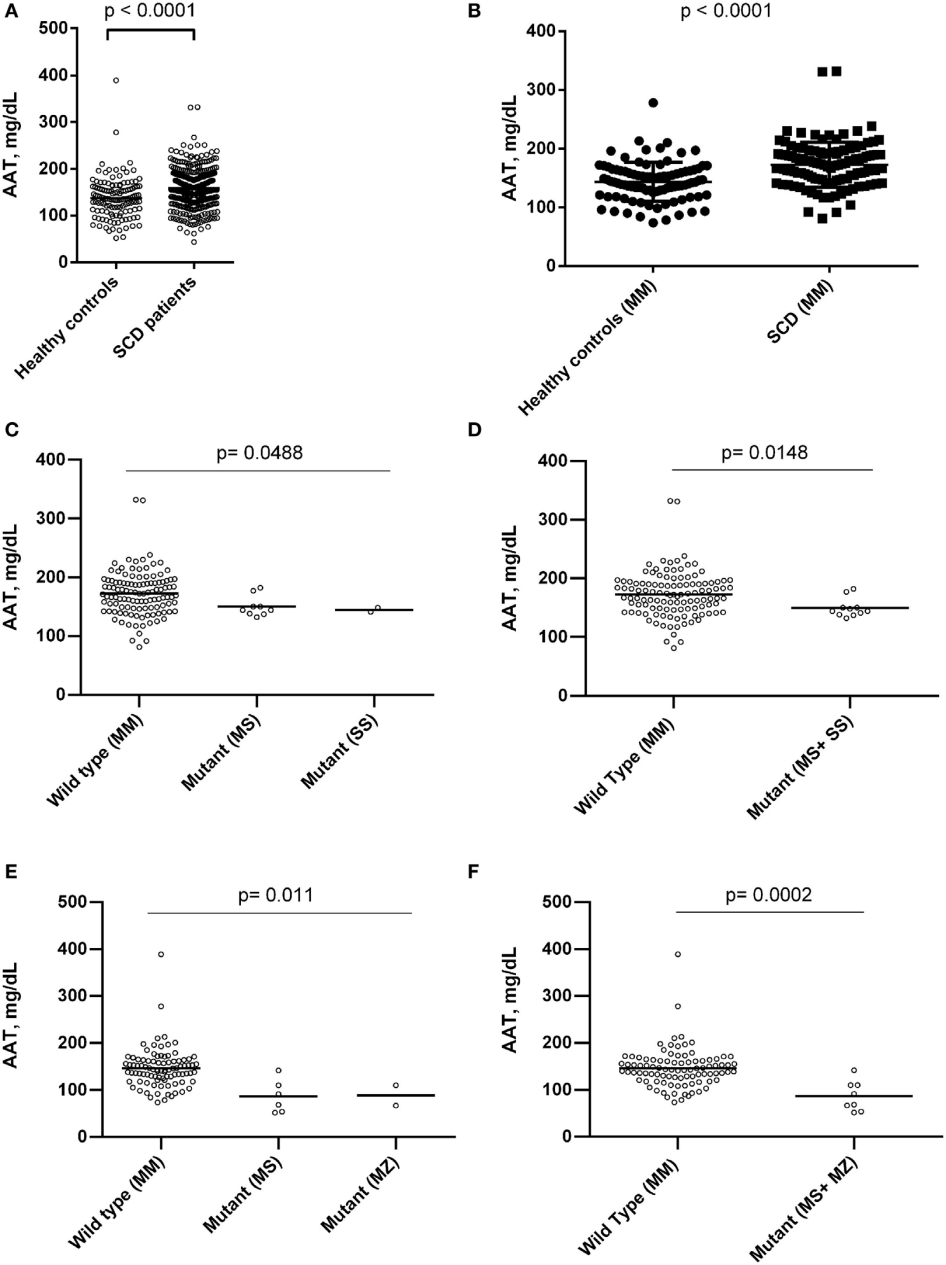
Association of alpha-1 antitrypsin (AAT) concentrations between healthy controls individuals and sickle cell disease (SCD) patients in steady state, and with mutations in *SERPINA1* gene between steady-state SCD patients and individuals of control group. **(A)** AAT levels between SCD patients and control group; **(B)** AAT levels between SCD patients and control group with proteinase inhibitor (PI)*MM genotype; **(C,D)** association of *SERPINA1* gene mutations and AAT concentration among SCD patients. SCD patients with genotype PI*MM had higher AAT levels than patients with mutant genotype analyzed separately (PI*MS and PI*SS) and together. **(E,F)** Association of *SERPINA1* gene mutations and AAT concentration among control group individuals. Control groups individuals with genotype PI*MM had higher AAT levels than individuals with mutant genotype analyzed separately (PI*MS and PI*MZ) and together.

Sickle cell disease patients presented higher levels of AAT when compared to controls (Figure 1). *SERPINA1* gene polymorphisms were analyzed among 126 SCD patients and 100 control group individuals. The PI*MM, or wild type, genotype was found in 115 (91.3%) of SCD patients and 92 (92%) controls; the PI*SS genotype was found in 2 (1.6%) SCD patients and no control individuals; the PI*MS genotype was found in 9 (7.1%) SCD patients and 6 (6%) controls; the PI*MZ genotype was found in just 2 (2%) controls. A comparison of AAT levels among the SCD patients and healthy controls showed higher levels among the patients, even when both groups were PI*MM. In addition, *SERPINA1* gene mutations have been associated with altered AAT levels (Figure 1).

### AAT Is Associated with Hematological and Biochemical Markers

**Figure 2:**
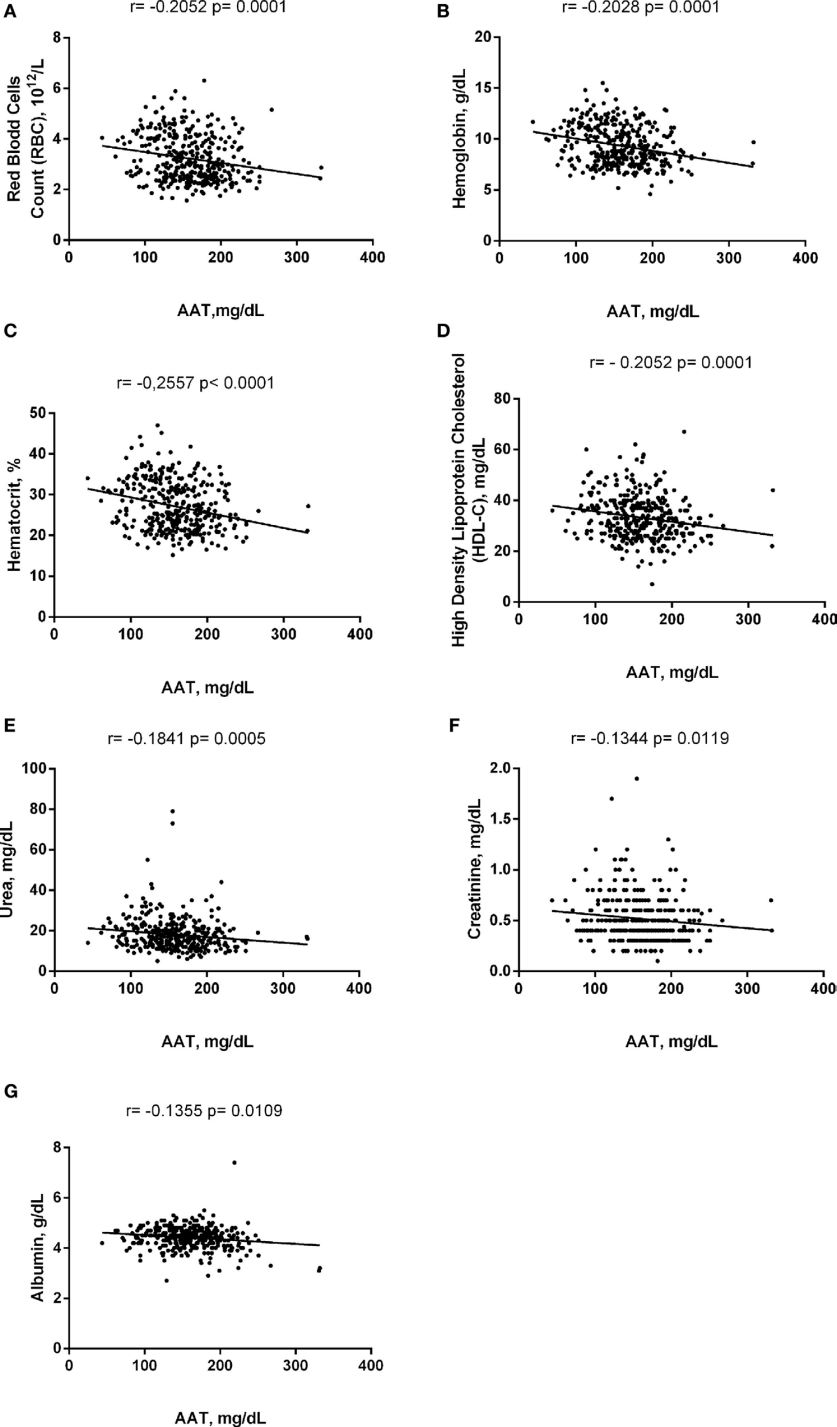
Negative correlations of alpha-1 antitrypsin (AAT) with laboratory parameters in steady-state sickle cell disease patients. **(A)** Red blood cells (RBCs); **(B)** hemoglobin; **(C)** hematocrit; **(D)** high-density lipoprotein of cholesterol (HDL-C); **(E)** urea; **(F)** creatinine; **(G)** albumin.

**Figure 3:**
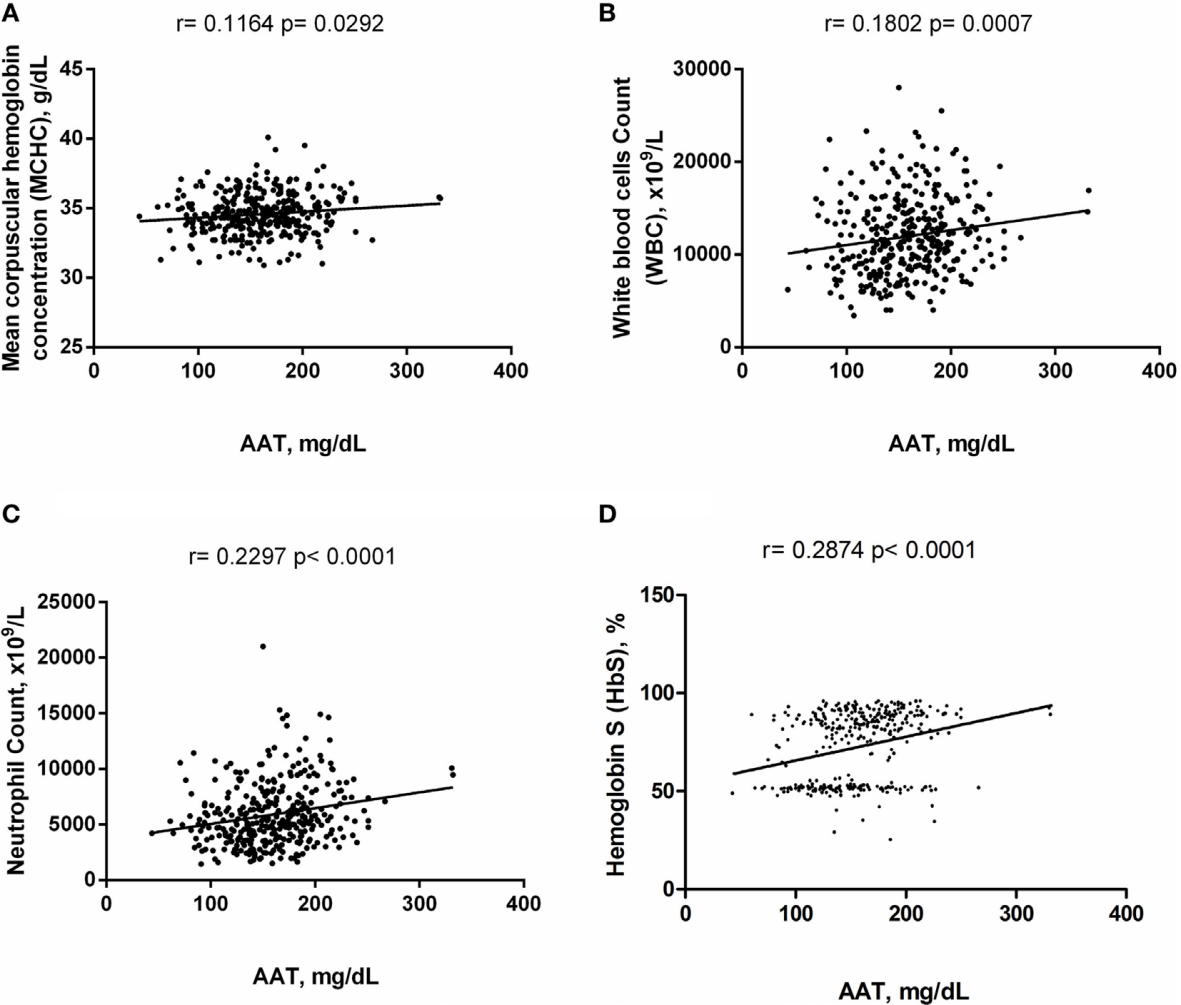
Positive correlations of alpha-1 antitrypsin (AAT) with hematological markers in steady-state sickle cell disease patients. **(A)** Mean corpuscular hemoglobin concentration; **(B)** white blood cell (WBC) counts; **(C)** neutrophil counts; **(D)** hemoglobin S (HbS).

**Figure 4:**
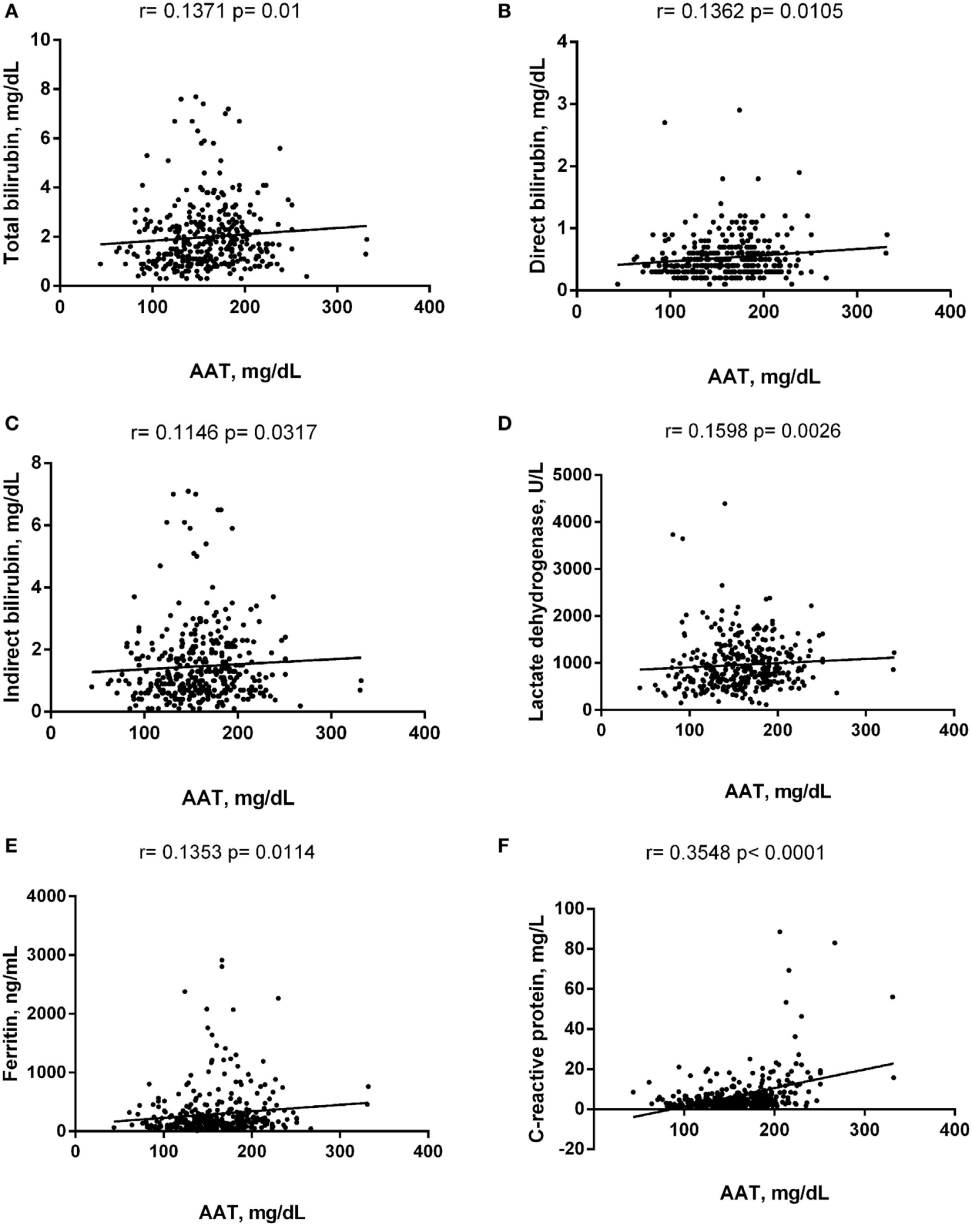
Positive correlations of alpha-1 antitrypsin (AAT) with biochemistry markers in steady-state sickle cell disease patients. **(A)** Total bilirubin; **(B)** direct bilirubin; **(C)** indirect bilirubin; **(D)** lactate dehydrogenase; **(E)** ferritin; **(F)** C-reactive protein.

**Figure 5:**
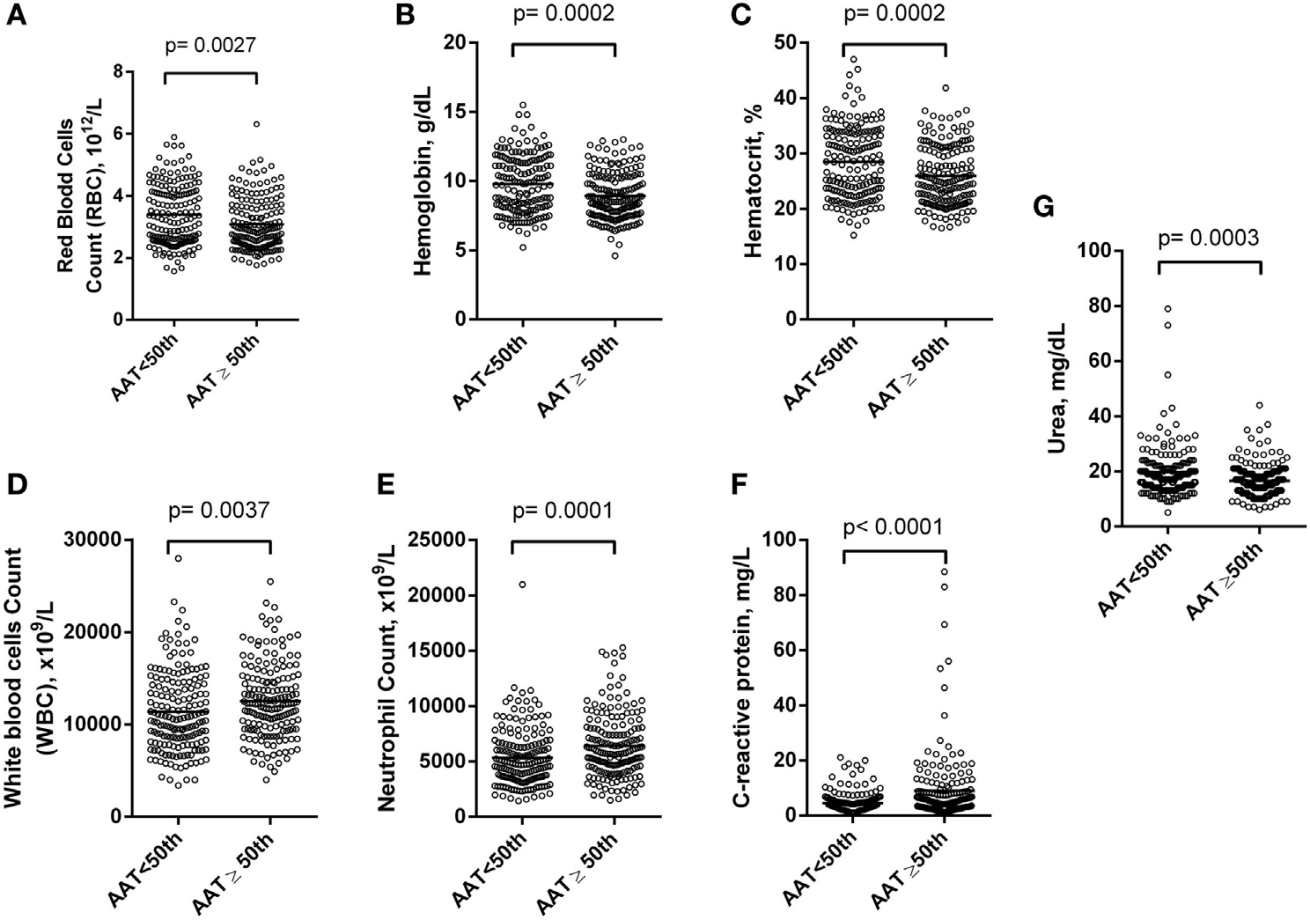
Association of hematological and chemistry markers in steady-state sickle cell disease (SCD) patients with alpha-1 antitrypsin (AAT) concentrations higher and lower than the 50th percentile. Statistical analyses indicate that SCD patients with alpha-1 antitrypsin concentration higher than the 50th percentile (158.0 mg/mL) had **(A)** lower count of red blood cells (RBCs); **(B)** lower concentration of hemoglobin (Hb); **(C)** lower concentration of hematocrit (Hct); **(D)** higher count of white blood cell (WBC); **(E)** higher count of neutrophils; **(F)** higher concentration of C-reactive protein (CRP); **(G)** higher concentration of urea.

Alpha-1 antitrypsin was found to be significantly negatively correlated with red blood cell (RBC) count, hemoglobin (Hb), hematocrit (Hct), high-density lipoprotein cholesterol (HDL-C), urea, creatinine, and albumin (Figure 2), yet positively correlated with mean corpuscular hemoglobin concentration, white blood cell (WBC) count, neutrophils, HbS (Figure 3), total bilirubin (BT), direct bilirubin (DB), indirect bilirubin (IB), lactate dehydrogenase (LDH), ferritin, and C-reactive protein (CRP) (Figure 4). This protein was also shown to be associated with some hematological and biochemical markers when present at concentrations higher and lower than the median value (158.0 mg/mL) described among SCD patients (Figure 5).

### AAT Is Associated with the Clinical Profiles of SCD Patients

**Figure 6:**
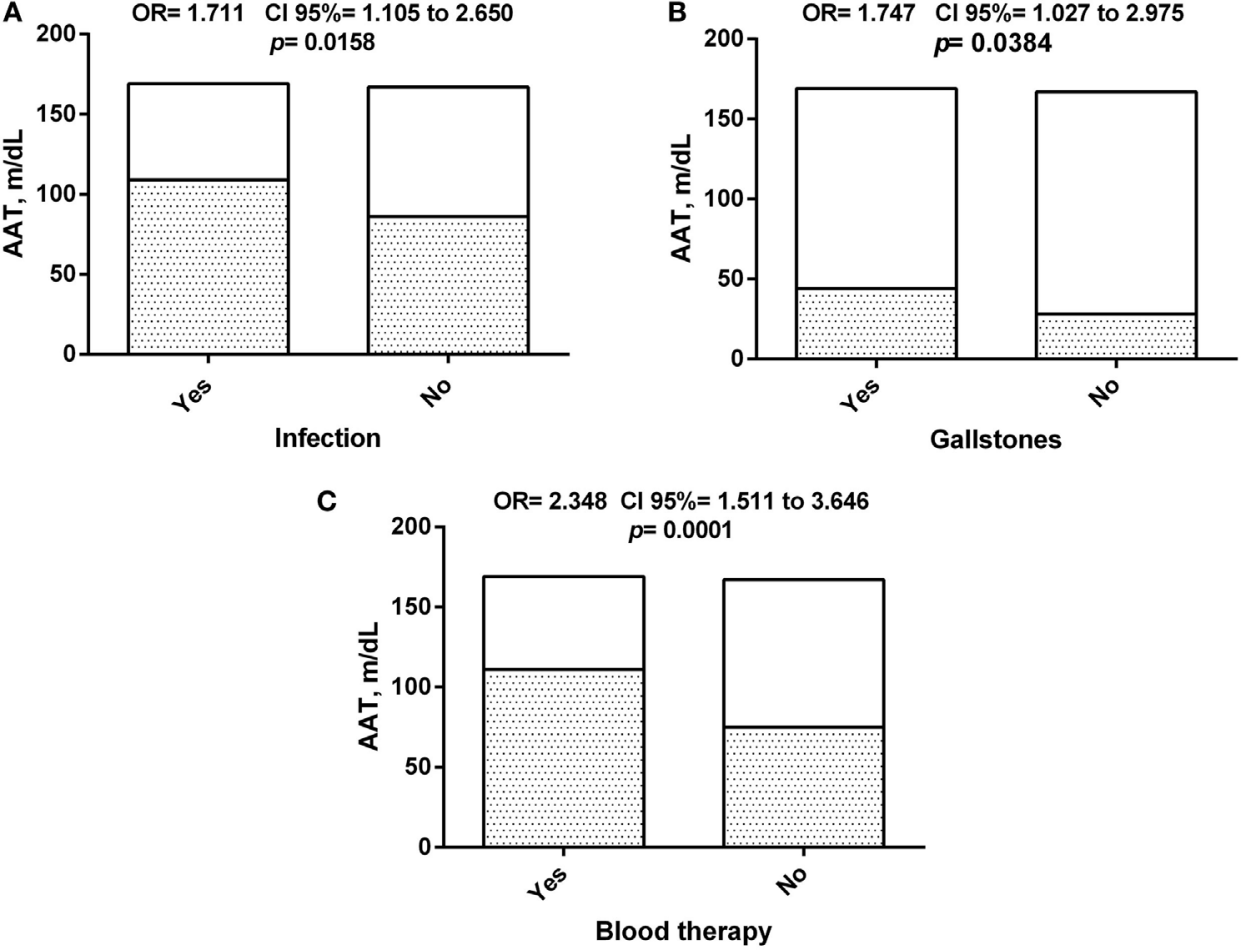
Association of alpha-1 antitrypsin (AAT) concentration between sickle cell disease (SCD) patients and clinical history. **(A)** Association of AAT concentration among SCD patients with history of infection episodes; **(B)** association of AAT concentration among SCD patients with history of gallstones; **(C)** association of AAT concentration among SCD patients with history of blood therapy.

Alpha-1 antitrypsin serum levels were found to be associated with some clinical outcomes related to SCD severity (Figure 6), with increased levels seen in patients that had two or more documented previous history of infection, occurrence of gallstones or received blood transfusion during follow-up. This seems to suggest the participation of this molecule in the clinical pattern of response to SCD.

## Discussion

Sickle cell disease is of epidemiological importance due to its high prevalence and high rates of morbidity and mortality worldwide. The clinical variability and the diversity of factors that modulate the disease remain poorly understood (2, 6, 7).

The present study found associations between serum levels of AAT (in both patients and controls) and the *SERPINA1* genotype, with higher AAT serum levels seen in wild type compared to mutant alleles. Elevated serum levels of AAT have been previously described in SCD (23). Likewise, a recent report has measured the complex neutrophil-derived azurophilic cytosolic protein elastase in complex with AAT and has found increased levels in SCD patients during crisis and ACS (28).

Considering the *SERPINA1* gene, it is known that the S and Z mutant alleles are most frequently found, which have been associated with reduced serum levels of AAT. AAT deficiency implies an increased risk of developing pulmonary emphysema and liver disease, as well as decreased immunomodulatory and anti-inflammatory capacity, as well as proteinase inhibitory properties (18, 27). In the liver, the presence of the Z allele may facilitate protein polymerization and hepatocyte accumulation secondary to altered AAT synthesis and chaperone binding induction (10, 29). Our results showed that approximately 10% of the SCD patients studied presented mutant alleles, although the vast majority had the (PI)*MM genotype. This indicates that both AAT levels and *SERPINA1* genotypes should be revisited as potential biomarkers that warrant further study in the context of SCD, as well as considered for future therapeutic applications, especially the treatment of pulmonary disease (30).

We found a significant positive correlation with serum bilirubin (TB, DB, and IB), LDH, ferritin and CRP, both, acute phases proteins. The present study demonstrated an association between high serum levels of AAT and more severe anemia, increased WBC and neutrophil counts, as well as altered CRP levels. Our findings are in agreement with previous reports describing the immunomodulatory and anti-inflammatory properties of AAT, which supports the notion that increased AAT levels prevents neutrophil adherence to the endothelium and reduces toll-like receptor expression and the production of pro-inflammatory cytokines, mainly in the lungs (17, 31, 32). CRP, a positive acute phase protein that rises rapidly during inflammatory processes, was shown to be positively correlated with AAT levels. CRP and AAT levels were also correlated in a previous report with SCD patients (23). Taking into account that CRP is an important inflammatory marker in clinical practice, due to easy of determining serum levels, as well as that AAT, an anti-inflammatory biomarker, is also relatively easy to quantify, it may be advisable to consider both together in clinical–epidemiological applications (33).

Increased AAT production, which occurs during the inflammatory response and in response to tissue damage, has been linked to liver dysfunction and with increased systemic levels of this protein (9, 10, 34). AAT plays an important role in leukocyte regulation, including the expression of surface molecules in monocytes and the inhibition of elastase and other serine proteinases produced by neutrophils. Accordingly, AAT controls tissue degradation promoted by proteases, especially neutrophil elastase, and inhibits the action of these pro-inflammatory enzymes on specific tissues, such as the effect of neutrophil elastase in lungs, with a described role on the COPD and emphysema (15–17, 20, 35–38).

Despite the proteinase inhibitory function of AAT, it also has an anti-inflammatory characteristics, with immunoregulatory activities against inflammatory blood cells, particularly, lymphocytes, monocytes, macrophages, and neutrophils, regulating the synthesis of leukotriene B4 (LTB4), an neutrophil chemoattractants, and of pro-inflammatory cytokines, such as interleukin (IL)-8, tumor necrosis factor alpha, IL-1, and IL-6; also, the role of the glycosylated and/or non-glycosylated forms of the AAT has been associated with its immunomodulatory and elastase inhibitory functions (39, 40). Interestingly, it was described a cytoprotective effect of AAT to blocking the neutrophil elastase accumulation in the renal tubules in experimental and human study of clinical acute kidney injury (41). Our results related to renal markers can support the hypothesis previously driven about the AAT as a biomarker of renal disease, and probably be of importance in the SCD patients, although it has not been studied yet with this focus, but we believe it will bring countless benefits for this group of patients.

Furthermore, the present study suggests that AAT plays an important role in the pathogenesis of SCD since the increased level of bilirubin is associated with hemolysis, and increased WBC count and CRP changes are associated with endothelial injury and the chronic inflammatory state described among SCD patients (7). Although AAT is a serine PI, we speculate that oxidative stress in SCD patients may lead to changes in the AAT molecule, favoring loss of its anti-inflammatory capacity, thus hindering its function of neutralizing inflammatory biological responses. With regard to hematological parameters, our results show significant negative correlation of AAT with RBC count, Hb, and Hct, thus strengthening the previously driven hypothesis (41). It is well known the role of the ischemia and reperfusion phenomenon and its association with altered metabolism and with tissue hypoxia and its contribution to increase the severity of several diseases, including the acute kidney injury, which is currently found among SCD (5, 42–44). Our finding related to the increased levels of the AAT concentration and its association with clinical symptoms, as well as the association with neutrophil counts, suggests that in the chronic inflammatory response presented by SCD disease patients, it may be an important marker of some specifics clinical pattern exhibited by these patients. The protease inhibitor properties, already described to AAT, may justify its increase among infection episodes; the gallstones occurrence may be related to its hepatic production and also with the hemolysis organ repercussion; the increase of blood therapy may represent the additional response of hemolysis, inflammation, and some immune system alteration among these patients, as well as the association of the disease severity (6).

In summary, SCD is associated with a heterogeneous clinical picture, with diverse clinical manifestations, with multiple organ damage (2, 6, 7, 45, 46). Thus, numerous factors may participate in the modulation of disease.

Our results reinforce the importance in research into prognostic markers in SCD, since they suggest a possible relationship between the levels of AAT and changes in its molecule as a result of oxidative stress and inflammation, and also suggests association of AAT levels with markers commonly investigated in the laboratory routine, with easy access for monitoring and estimation of severity of the disease.

Genotypes in the *SERPINA1* gene described in this study corroborate the presence of deficient AAT production. Our results suggest a direct correlation between AAT and SCD clinical manifestations and pathology—hemolytic, inflammatory, and endothelial injury. This hypothesis deserves further studies that should also focus on the interaction of mutations in the *SERPINA1* gene and AAT molecule changes and their involvement in the immune response. The role of oxidative stress of SCD on the AAT molecule may also prove quite worthwhile.

## Ethics Statement

The present study received approval from the Institutional Review Board of the Gonçalo Moniz Institute of the Oswaldo Cruz Foundation (IGM-FIOCRUZ), and each included study subject’s legal guardian agreed to participation and biological sample collection. This study followed the ethical guidelines established by the Declaration of Helsinki, as well as its subsequent revisions, and informed written consent was obtained from each control subject and SCD patient’s guardian. When applicable, the children’s acceptance was registered.

## Author Contributions

MSG and MOSC conceived and designed the study; MBC, ALCSS, and APASP performed laboratorial analysis; LCR and VMLN attended patients; MSG, MOSC, MBC, and APASP analyzed the data; CCG, CVBF, AA, and RPS critically revised the manuscript; MSG contributed reagents/ materials/analysis tools; MSG, ALCSS, and MOSC wrote the manuscript; MOSC, ALCSS, MBC, APASP, LCR, VMLN, CVBF, CCG, RPS, AA, and MSG reviewed and approved the manuscript final version.

## Conflict of Interest Statement

The authors declare that the research was conducted in the absence of any commercial or financial relationships that could be construed as a potential conflict of interest.
